# DNA-Based Taxonomy in Ecologically Versatile Microalgae: A Re-Evaluation of the Species Concept within the Coccoid Green Algal Genus *Coccomyxa* (Trebouxiophyceae, Chlorophyta)

**DOI:** 10.1371/journal.pone.0151137

**Published:** 2016-03-30

**Authors:** Veronica Malavasi, Pavel Škaloud, Fabio Rindi, Sabrina Tempesta, Michela Paoletti, Marcella Pasqualetti

**Affiliations:** 1 Interdepartmental Center of Environmental Science and Engineering (CINSA), University of Cagliari, Cagliari, Italy; 2 Department of Botany, Faculty of Science, Charles University of Prague, Prague, Czech Republic; 3 Dipartimento di Scienze della Vita e dell’Ambiente, Università Politecnica delle Marche, Ancona, Italy; 4 Department of biological and ecological sciences, Tuscia University, Viterbo, Italy; Consiglio Nazionale delle Ricerche (CNR), ITALY

## Abstract

*Coccomyxa* is a genus of unicellular green algae of the class Trebouxiophyceae, well known for its cosmopolitan distribution and great ecological amplitude. The taxonomy of this genus has long been problematic, due to reliance on badly-defined and environmentally variable morphological characters. In this study, based on the discovery of a new species from an extreme habitat, we reassess species circumscription in *Coccomyxa*, a unicellular genus of the class Trebouxiophyceae, using a combination of ecological and DNA sequence data (analyzed with three different methods of algorithmic species delineation). Our results are compared with those of a recent integrative study of Darienko and colleagues that reassessed the taxonomy of *Coccomyxa*, recognizing 7 species in the genus. Expanding the dataset from 43 to 61 sequences (SSU + ITS rDNA) resulted in a different delimitation, supporting the recognition of a higher number of species (24 to 27 depending on the analysis used, with the 27-species scenario receiving the strongest support). Among these, *C*. *melkonianii* sp. nov. is described from material isolated from a river highly polluted by heavy metals (Rio Irvi, Sardinia, Italy). Analyses performed on ecological characters detected a significant phylogenetic signal in six different characters. We conclude that the 27-species scenario is presently the most realistic for *Coccomyxa* and we suggest that well-supported lineages distinguishable by ecological preferences should be recognized as different species in this genus. We also recommend that for microbial lineages in which the overall diversity is unknown and taxon sampling is sparse, as is often the case for green microalgae, the results of analyses for algorithmic DNA-based species delimitation should be interpreted with extreme caution.

## Introduction

Species are one of the fundamental units of biology, making them comparable in importance to genes, cells, and organisms, some of the fundamental units at lower levels of biological organization [1]. Species delimitation is essential for our understanding of ecosystems and biodiversity, which is necessary for effective decision making in conservation planning [2]. In the practice, however, defining species boundaries is often far from straightforward. In the past this process has been complicated by the application of many different species concepts and has been strongly influenced by new types of data that have become available in time with technological and methodological advances. For certain groups of organisms, establishing species boundaries has proved particularly challenging; green microalgae (small-sized algae belonging to the Chlorophyta and Streptophyta lineages, Leliaert et al.[3]) can certainly be included in this category.

For a long time, the classification of these organisms has been entirely based on morphological and cytological features of vegetative stages in their life cycle [4]. Due to their simple morphology, however, most green microalgae offer a very limited set of characters that may be useful for this purpose. It is also known that some of these characters are affected by considerable phenotypic plasticity and can vary substantially under different environmental conditions, with the risk of misinterpretations when samples from different habitats or grown in different conditions are examined [4,5,6]. Molecular studies have drawn a scenario in sharp contrast with morphology-based classifications, highlighting that for these algae the morphological species concept is burdened with a high degree of misinterpretation [7,8]. In particular, molecular evidence has unravelled rampant morphological convergence in the evolution of these organisms, demonstrating the existence of cryptic species within many taxa defined on morphological basis [2,9,10,11,12,13]. New species of microchlorophytes are now described and separated from congeners combining morphological observations with molecular data, either as phylogenetic analyses of DNA sequence data and/or secondary structure of ribosomal DNA [14,15,16].

In the last decade, there have been numerous attempts to define species algorithmically and many new methods for delimitation of species based on DNA sequences have been proposed [17,18,19]. These methods appear to have considerable potential, and their strengths and limitations have been extensively discussed. The most popular method currently used to delimit species boundaries is probably the generalized mixed Yule-coalescent model (GMYC), attempting to find the transition point between species-level and population-level variability based on a shift in the rate of an ultrametric tree branching [20]. Quite recently, a number of tree-based species discovery methods have been introduced, including the promising Poisson tree process (PTP) models. In contrast to GMYC, PTP models delimit species in terms of the number of substitutions, thereby they are not depending on the accuracy of ultrametric tree estimations. In addition, they may outperform other species delimitation methods when evolutionary distances are small [19]. Finally, several models use a Bayesian modelling approach to generate the posterior probabilities of species assignments taking account of uncertainties due to unknown gene trees and the ancestral coalescent process [21], including the bPTP [22], BAPS [23], STRUCTURAMA [24], BP&P [21], and STACEY [25]. Some of these methods have been applied in recent studies on green microalgae (e.g., Sadowska-Des et al.[13], Darienko et al.[4]). So far, however, their use has been limited to a few taxa and the applicability of their assumptions to many groups of microchlorophytes needs to be verified with additional work.

In this study, based on the examination of a new strain from an extreme habitat, we reconsider species delimitation in *Coccomyxa*, a widespread unicellular genus belonging to the class Trebouxiophyceae. The genus has a cosmopolitan distribution and is well known for its ecological versatility, as it includes species found in many aquatic and terrestrial habitats, both free-living and symbionts [26,27,28], including sites with extreme environmental traits [29]. Species of *Coccomyxa* are characterized by a small size (6–14 x 3–6 μm), an irregular elliptical to globular cell shape, a parietal chloroplast without a pyrenoid and the absence of any flagellated stages [4]. Due to the scarcity of morphological characters useful for taxonomic purposes, *Coccomyxa* represents a good example of the problems highlighted above; species delimitation in this genus (and its separation from the similar genera *Pseudococcomyxa* and *Choricystis*) using only morphological features has been traditionally problematic. Recently Darienko et al. [4] reassessed the taxonomy of *Coccomyxa* based on an integrative approach combining morphological, physiological and molecular data for 41 strains. These authors subdivided the genus in 7 species based primarily on analyses for species delimitation (ABGD, K/θ, GMYC, PTP) performed on ribosomal DNA and an ITS2 CBC/HCBC approach. Here, we re-examine the taxonomy of this genus expanding the dataset of Darienko et al. [4] with a number of additional sequences not used by these authors. We show that analyses for species delimitation based on this expanded database lead to a major advancement in the classification of this genus, supporting an alternative scenario with a considerably higher number of species. We argue that ecological characters are more important than morphological characters and we describe *Coccomyxa melkonianii* sp. nov., for a strain isolated from a river strongly contaminated by heavy metals.

## Materials and Methods

### Ethics and data availability statement

The field sampling was done in agreement with IGEA S.p.A. (In-House Company of the Autonomous Region of Sardinia), a legal entity in the activity for the safety, environmental restoration and reclamation of the investigated mining areas. No specific permission was required for the sampled locations as we were collecting water samples on public lands. The field studies did not involve endangered or protected species. All relevant data are within the paper and its S1–S6 Datasets.

### Study area and sampling

The strain of *Coccomyxa* described as a new species in this study was isolated from the Rio Irvi, a river located in the south-western part of Sardinia, Italy. The Rio Irvi river is about 7 km long and flows in the Arburese mining district (SW Sardinia), where the Ingurtosu lead and zinc sulphide ore deposits were mined until 1968. This river is characterized by a pH ranging between 3 and 7, and a high content of heavy metals (Cd, Co, Fe, Mn and Zn), particularly iron, documented by the bright red colour of the water throughout most of its length.

**Fig 1:**
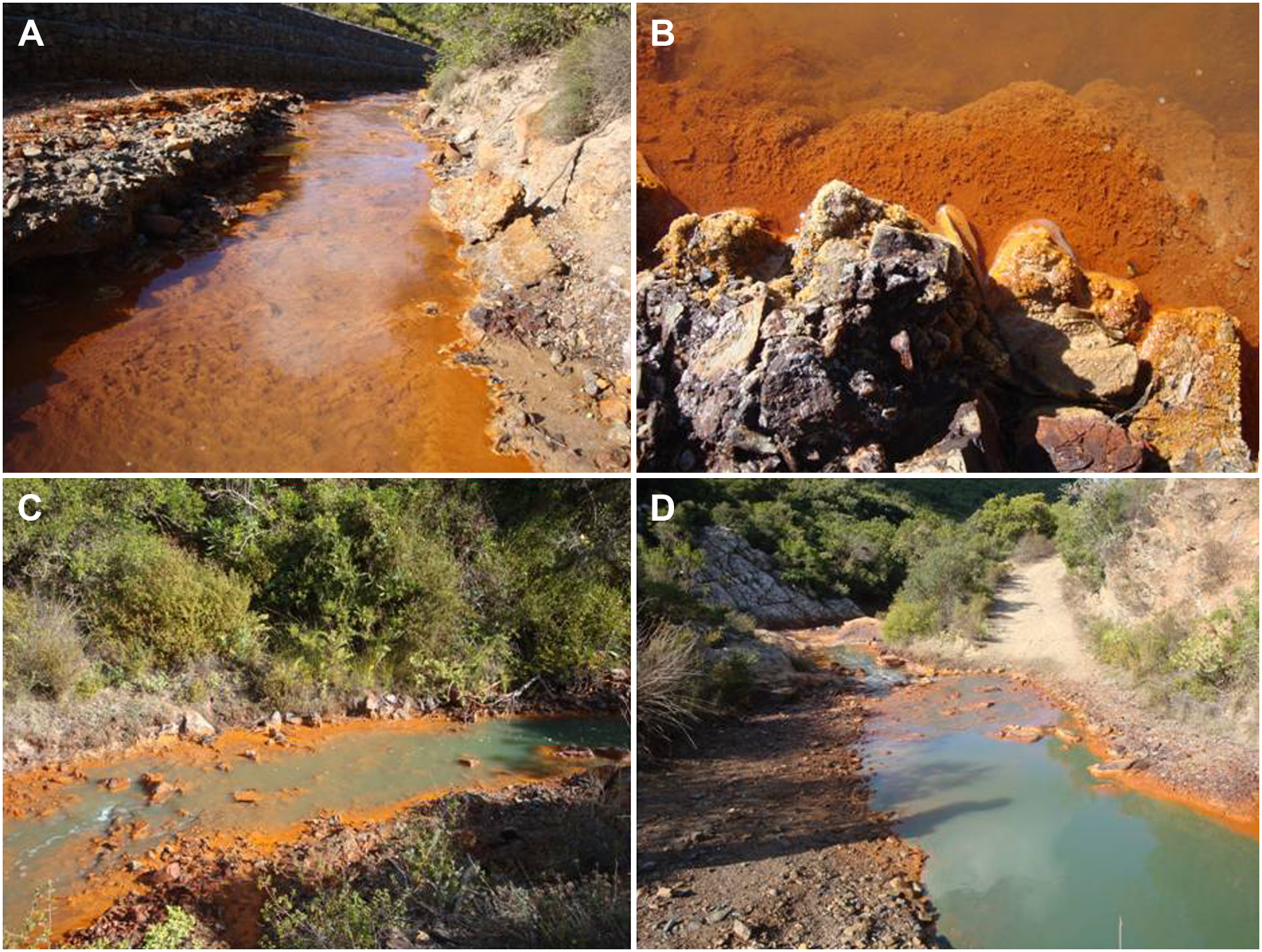
The Rio Irvi river, Locality Montevecchio-Ingurtosu, Sardinia, Italy. **A**. The river base covered by iron sediments, **B**. The sampling site of *Coccomyxa* SCCA 048, **C-D**. Artificial colouring of the river water caused by high concentrations of Cd, Co, Zn and Fe heavy metals.

A standard operating procedure that applies to the collection of the algae from freshwater and terrestrial habitats was used. The alga was found in a station (N 39.546894, E 8.480278) where pH of the water was 6.85. The collection was made by scraping reddish ferrous material from rocks present at the river's edge (Fig 1). The physico-chemical parameters measured in the station are given in Table 1.

**Table 1:** Physico-chemical parameters and metal concentration of water at the station in Rio Irvi where *Coccomyxa melkonianii* SCCA 048 was collected.

Parameter	Value
Water temperature	21.6°C
pH	6.85
EH	-14.20 mV
Cd	1.43 mg/L
Co	1.75 mg/L
Total Fe	226.82 mg/L
Mn	81.18 mg/L
Ni	2.97 mg/L
Pb	0.003 mg/L
Zn	956.60 mg/L
SO_4_	3694 mg/L

### Isolation and growing conditions

The collected samples were stored in 150 mL plastic flasks. The green alga examined in this study was isolated by glass—capillaries [30] with an Olympus CKX41 (Olympus, Tokyo, Japan) inverted light microscope. After isolation, stock cultures were established under controlled laboratory conditions (25°C, 12:12 h L:D, 80–100 μmol photons·m^-2^·s^-1^). The alga was maintained in modified WARIS-H culture medium [31] without soil extract under cool luminescent white light.

### Documentation / observation

*Coccomyxa* SCCA048 was investigated using an Olympus CKX41 light microscope. An Olympus LCmicro camera imaging software was used to obtain morphometric measurements. For transmission electron microscopy (TEM) investigations, samples were fixed with 2% paraformaldehyde + 2.5% glutaraldehyde in cacodylate sucrose buffer (0.1 M cacodylate, 0.09 M sucrose, 0.01 M CaCl_2_, 0.01 M MgCl_2_, pH 6.9) containing 0.075% ruthenium red and 0.075 M lysine acetate for 20 min at 4°C. Samples were washed in the same buffer containing 0.075% ruthenium red and then fixed with 2% paraformaldehyde + 2.5% glutaraldehyde in cacodylate sucrose buffer containing 0.075% ruthenium red overnight at 4°C. After washing in the same buffer containing 0.075% ruthenium red, samples were post-fixed in 1% OsO_4_ + 0.075% ruthenium red in 0.1 M cacodylate buffer, pH 7.2, for 1 hour at room temperature and then washed again in the same buffer containing 0.075% ruthenium red. Specimens were dehydrated in a graded ethanol series and embedded in LRWhite resin (Multilab Supplies, Surrey, England). The resin was polymerised in tightly capped gelatine capsules for 48 h at 50°C. Thin sections were cut with Reichert Ultracut and LKB Nova ultramicrotomes using a diamond knife, collected on copper grids, stained with uranyl acetate and lead citrate, and observed with a JEOL 1200 EX II electron microscope at 100kV. Micrographs were acquired by an Olympus SIS VELETA CCD camera equipped with iTEM software.

### DNA extraction, gene amplification and sequencing

Genomic DNA was extracted using the Maxwell^®^ 16 Automated DNA Purification Instrument (Promega) using the cartridges “LEV (Low Elution Volume) Plant DNA Kit” (Promega) in a final volume of 50 μl of Nuclease-Free Water (provided in the kit).

The gene of the small subunit of the nuclear ribosomal DNA (SSU rDNA) and the internal transcribed spacer region (ITS1-5.8S-ITS2) of the rDNA were amplified by PCR. The amplification was carried out in a final volume of 25 μl, in a mixture containing 2 μl of extracted DNA, 0.5 μl of each primer 10 mM, 2.5 μl of MgCl_2_ 25mM (Promega), 1.5 μl of 5x buffer (Promega), 0.5 μl of dNTPs 10 mM, and 5 U of Go-Taq Polymerase (Promega); the final volume was reached adding ultrapure deionized water. The SSU rDNA gene (2217 bp) was amplified using the primers SSU1F (5’-TGG TTG ATC CTG CCA GTA G-3’) and SSU2R (5’-TGA TCC TTC CGC AGG TTC AC-3’; [32]). In addition, a new couple of primers was designed to amplify the central section of the same gene; the primers used were NewcocSSUF (5’-GTC GGC GAG GTT ACT TTG AG-3’) and NewcocSSUR (5’-ACC GGA ATC AAC CTG ACA AG-3’). The PCR amplification was carried out using the following condition: 3 min at 95°C, followed by 40 cycles of 30 sec at 95°C, 30 sec at 54°C, 2 min at 72°C, and a final extension of 7 min at 72°C [32]. For the ITS rDNA region (ITS1-5.8S-ITS2), 641 bp were amplified using the primers ITS5 (5’-GGA AGT AAA AGT CGT AAC AAG G-3’) and ITS4 (5’-TCC TCC GCT TAT TGA TAT GC-3’; [33]). In this case the PCR protocol consisted of 3 min at 95°C, followed by 30 cycles of 30 sec at 95°C, 30 sec at 54°C, 45 sec at 72°C, and a final extension of 10 min at 72°C. The DNA sequence of the newly obtained 18S-ITS1-5.8S-ITS2 rRNA gene region has been deposited in the GenBank database under accession no. KU696488.

### Alignments and phylogenetic analyses

The SSU and ITS sequences of *Coccomyxa* SCCA048 were added to the alignment of 43 sequences provided by Darienko et al. [4] as a S2 Dataset (comprising 41 ingroup and 2 outgroup taxa). In addition, BLAST searches against various ITS rDNA *Coccomyxa* sequences revealed the existence of 54 closely related sequences not included in the original alignment, which were also added to the dataset (S1 Table). If available, the newly included ITS rDNA sequences were concatenated with SSU rDNA sequences originated from the same organisms. The SSU rDNA sequences were aligned using the MAFFT v. 6 software [34] under the Q-INS-I strategy, and checked for obvious sequencing errors. The ITS rDNA sequences were first aligned according to their secondary structure by comparison of the ITS2 structures and alignments provided by Darienko et al. [4](S1 Dataset). Since the ITS rDNA sequences were very divergent and obviously saturated, and since their alignment was ambiguous even based on the ITS1+2 secondary structures, we eliminated ambiguously aligned positions using the program Gblocks v. 0.91b [35], allowing all options for a less stringent selection. The alignment was reduced to 58% of the original 917 positions. SSU and ITS rDNA sequences were then concatenated, and 14 alignment positions containing ambiguous bases were deleted. The resulting alignment was 2,311 bp long, consisting of 98 taxa (96 ingroup and 2 outgroup sequences; S1 Table). The alignment was partitioned into SSU rDNA, ITS1, 5.8S rDNA, and ITS2 regions. For each of the alignment partitions, the most appropriate substitution model was estimated using the Bayesian information criterion (BIC) as implemented in jModelTest 2.1.4 [36]. This BIC-based model selection procedure selected the following models: (1) TIM1ef + I + Γ for SSU rDNA, (2) TIM2 + Γ for ITS1 and ITS2, and (3) K80 for 5.8S rDNA.

For the purpose of phylogenetic analyses, the alignment was reduced to 62 taxa (60 ingroup and 2 outgroup sequences) for which both SSU and ITS rDNA sequences were available (S2 Dataset; S1 Table). The identical sequences were then merged to speed-up the analyses. A phylogenetic tree was inferred by Bayesian inference (BI) using MrBayes version 3.2.6 [37]. The analysis was carried out on partitioned datasets using the different substitution models selected by jModelTest 2.1.4. We used the GTR model of nucleotide substitution instead of TIM, given that it was the best matching model available in MrBayes. All parameters were unlinked among partitions. Two parallel Markov chain Monte Carlo (MCMC) runs were carried out for eight million generations, each with one cold and three heated chains. Trees and parameters were sampled every 100 generations. Convergence of the two cold chains was assessed during the run by calculating the average standard deviation of split frequencies (SDSF). The final SDSF value was 0.00098. Finally, the burn-in values were determined using the ‘sump’ command. Bootstrap analyses were performed by maximum likelihood (ML) and weighted maximum parsimony (wMP) criteria using GARLI, version 2.01 [38] and PAUP*, version 4.0b10 [39], respectively. ML analyses consisted of rapid heuristic searches (100 pseudo-replicates) using automatic termination (genthreshfortopoterm command set to 200,000; stopgen and stoptime commands set to 10,000,000, respectively). The analyses were performed on partitioned datasets using the different substitution models selected by jModelTest 2.1.4. The wMP bootstrapping (1,000 pseudo-replicates) was performed using heuristic searches with 100 random sequence addition replicates, tree bisection reconnection swapping, random addition of sequences, and gap characters treated as missing data. Character weights were assigned using the rescaled consistency index on a scale of 0 to 1,000. New weights were based on the mean fit values for each character over all trees in the memory.

### DNA-based species delimitation

Three different approaches were used to infer putative species boundaries based on DNA sequence data: the most commonly used GMYC analysis, a Bayesian implementation of PTP approach (bPTP; [22]), and the STACEY analysis with *a priori* selected species scenarios, evaluated by the SpeciesDelimitationAnalyser tool. All analyses were performed on four different datasets. First, we investigated the concatenated SSU + ITS rDNA (62 taxa) dataset and the ITS rDNA (98 taxa) dataset (S1 Table). Second, two different ITS rDNA alignments were analysed in both the 62- and 98-taxa datasets: the original, not trimmed alignment of 917 nucleotide positions; and the reduced alignment with ambiguously aligned positions eliminated by the program Gblocks, which consisted of 528 nucleotide positions. All datasets are provided as S3, S4, S5 and S6 Datasets.

In the GMYC approach, Bayesian analyses were performed with BEAST 1.8.2 [40] to obtain ultrametric trees under the assumption of uncorrelated lognormal relaxed molecular clock. The analyses were performed on partitioned datasets using the different substitution models described above and under the constant population size coalescent as the tree prior. Ucld mean prior was set to exponential distribution with mean 10 and initial value 1. Five MCMC analyses were run for 30 million generations, sampling every 10,000 generations. The outputs were diagnosed for convergence using TRACER v. 1.6 [41] and the five tree files were merged using the burn-in set to 3 million generations. Consensus trees were generated using TreeAnnotator 1.8.2. GMYC analyses were performed on consensus trees under the single-threshold model, using the SPLITS package [42] in R 3.1.3 [43].

The bPTP analyses were conducted on the bPTP web Server (http://species.h-its.org/ptp/) based on unrooted phylogenetic trees inferred by MrBayes. The analyses were run for 200,000 generations. Finally, the Bayesian posterior probabilities of different species scenarios were estimated using STACEY as implemented in BEAST 2.3.0 [44], using a Relaxed Clock Log Normal clock model. The analyses were run for 10 million generations on partitioned datasets using the different substitution models described above. Cluster analyses were performed using SpeciesDelimitationAnalyser [45], with a burnin set to 20% and the height below which nodes get collapsed adjusted to 0.00001. All the Bayesian analyses were run using the resources available from the CIPRES Science Gateway [46].

### Analyses of ecological data

The following information was collected for all investigated taxa with known ecological data (S1 Table): living stage (free living, lichen photobiont, ciliate symbiont), habitat (water, soil, rock, bark), and resilience (mesophilic, extremophilic). Data were collected based on the information provided on web pages of algal culture collections hosting the investigated strains, as well as searching publications [47,48] and lichen herbarium databases (http://lichenportal.org/portal/). If not specified, habitat of lichens harbouring *Coccomyxa* was determined by known ecological preferences of particular taxa.

Two approaches were applied to assess the effect of different species scenarios on ecological differentiation of particular species. First, chi-square tests were performed to assess the statistical significance of differences in ecological traits between the putative species. However, since the species scenarios also included those species composed of a few, or even a single taxon, chi-squared approximations could be biased due to the small cell values in the contingency tables. Therefore, we additionally used non-parametric Mantel tests to assess the significance of the correlation between the patristic and ecological distance matrices. Under every species scenario, each putative species was characterized by a single representative taxon and a set of ecological trait frequencies calculated from all taxa assigned to the particular species. All computations were performed in R 3.1.3. Patristic distance matrices were calculated with the *cophenetic* function in the R-package ape [49] from the ultrametric phylogenetic trees. Ecological distance matrices (i.e. ecological dissimilarities) were computed based on the Euclidean metric with the *dist* function. Mantel tests were performed with the *mantel*.*rtest* function in the R-package ade4 [50].

To visualize ecological preferences of particular *Coccomyxa* species, we mapped the evolution of ecological traits onto the ultrametric phylogenetic tree generated by the GMYC analysis of ITS rDNA dataset comprising 98 taxa and 528 nucleotide positions. Ancestral trait reconstructions were inferred using the ACCTRAN parsimony method with the *anc*.*acctran* function in the R-package phangorn [51].

### Nomenclature

The electronic version of this article in Portable Document Format (PDF) in a work with an ISSN or ISBN will represent a published work according to the International Code of Nomenclature for algae, fungi, and plants, and hence the new names contained in the electronic publication of a PLOS article are effectively published under that Code from the electronic edition alone, so there is no longer any need to provide printed copies. The online version of this work is archived and available from the following digital repositories: PubMed Central, LOCKSS.

## Results

### Light and electron microscopy of the newly isolated strain SCCA048

**Fig 2:**
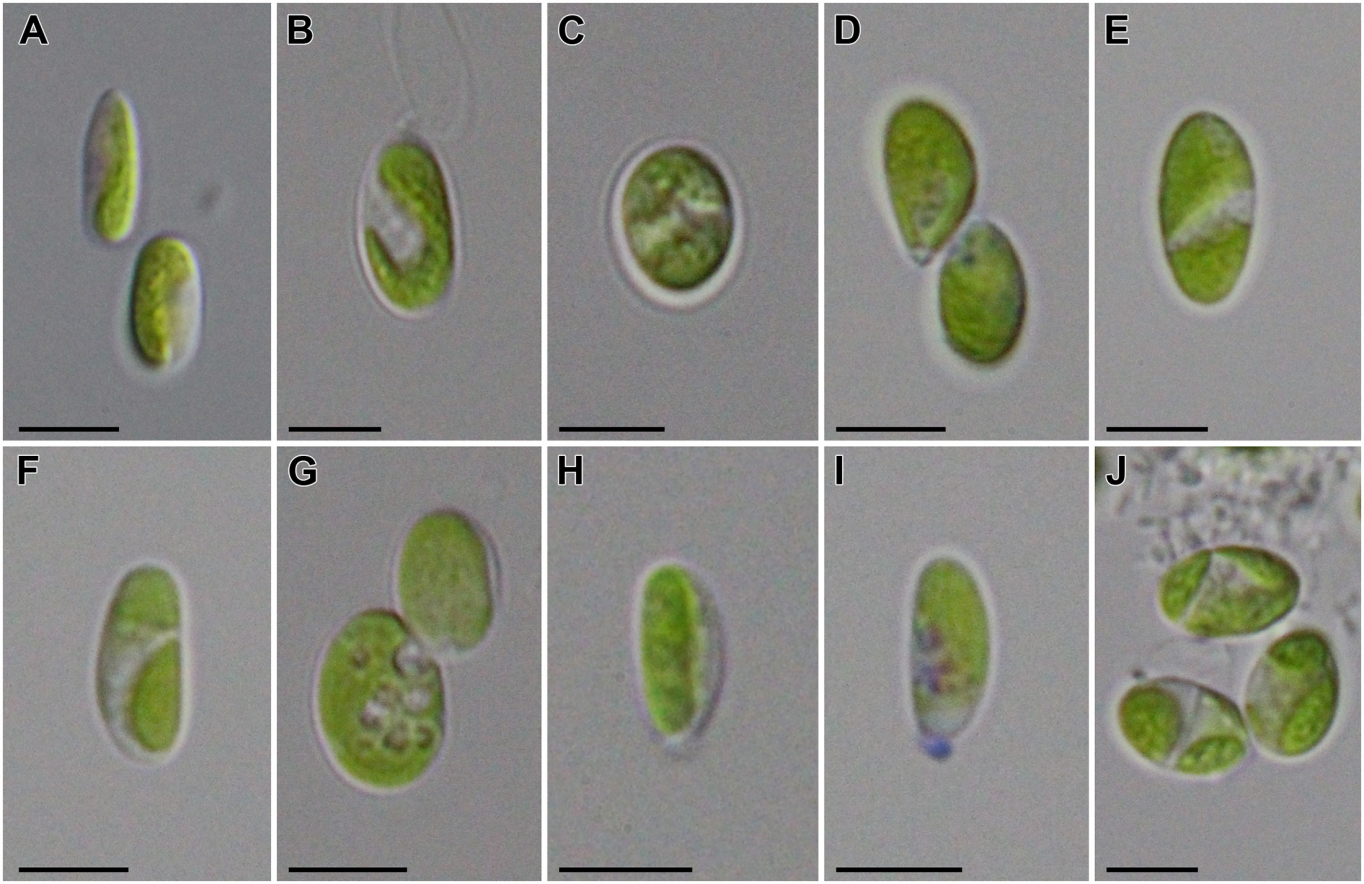
Morphology of *Coccomyxa melkonianii* SCCA048. **A-D**. Morphological plasticity of vegetative cells, **E-F**. Cells containing two plastids, **G**. Accumulation of lipid droplets in the cytoplasm, **H-I**. Formation of small mucilaginous caps, **J**. Mature cells (note the autospore formation at the bottom left cell). Scale bars = 5 μm.

Cells of the strain *Coccomyxa* sp. SCCA048 were morphologically highly variable (Fig 2), exhibiting elongated cylindrical (Fig 2A), ovoid (Fig 2B), broadly ellipsoidal (Fig 2C), and drop-like shapes (Fig 2D). They usually contained a single parietal chloroplast (Fig 2B and 2D), though two chloroplasts were rarely observed (Fig 2E and 2F). Mature cells were often filled by lipid droplets of various size (Fig 2G). Mucilaginous colonies were never formed, although cultivated in either liquid or agar-solidified media. Small mucilaginous caps were, however, observed in a few cells (Fig 2H and 2I). Asexual reproduction took place by cell division and a formation of 2–4 autospores (Fig 2J), released by rupture of the cell wall at the rounded apex of the cell.

**Fig 3:**
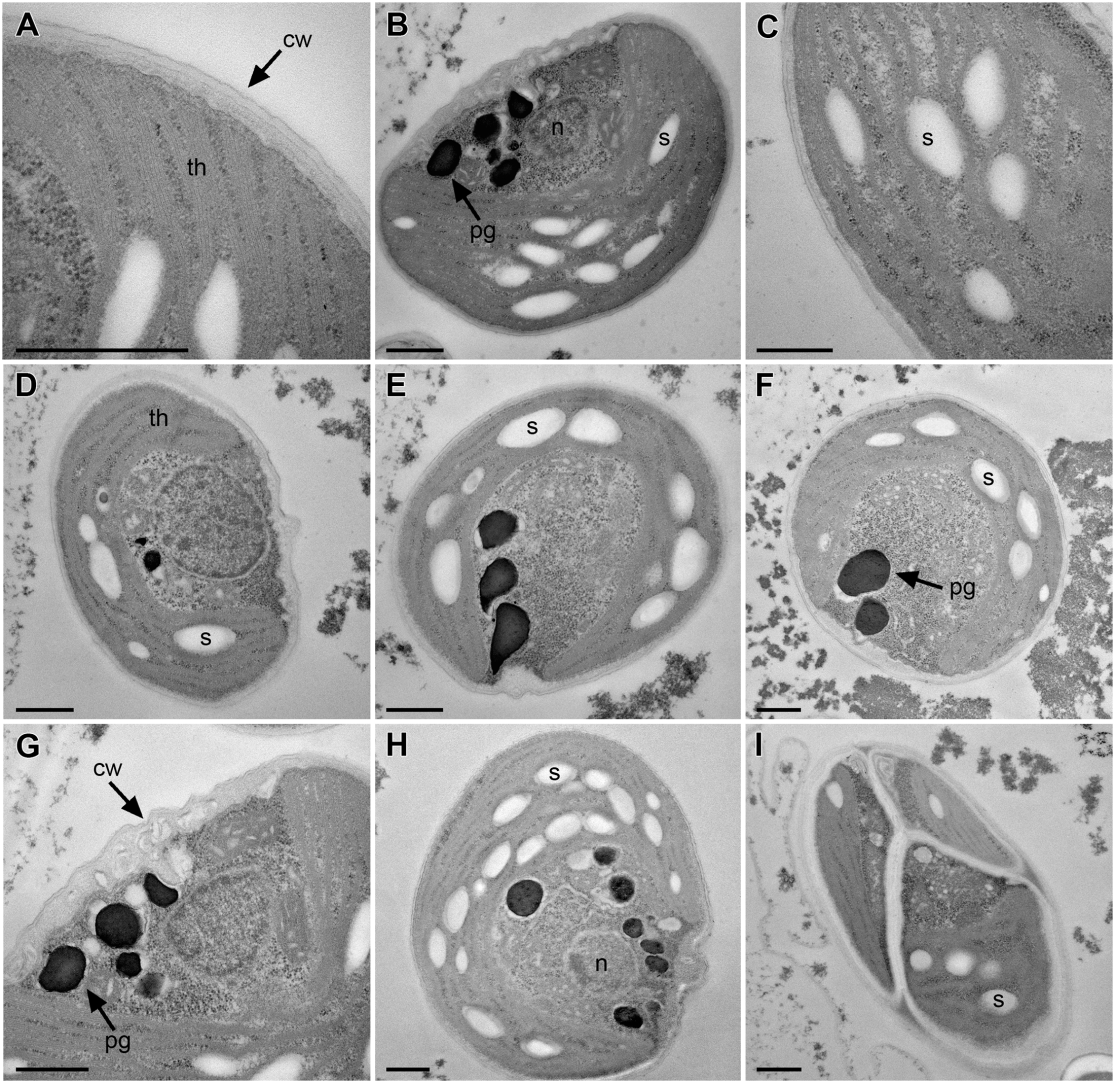
Ultrastructure of *Coccomyxa melkonianii* SCCA 048. **A**. Trilaminar cell wall, **B**. Cup-shaped chloroplast. Note the laterally positioned nucleus, **C**. Chloroplast ultrastructure. Note the absence of pyrenoid and the presence of distinct starch grains, **D-F**. Cells with variously arranged cup-shaped chloroplasts, **G**. A detail of nucleus-containing cytoplasm, **H**. Vegetative cell. Note a number of starch grains (electron-lucent) and plastoglobuli (electron-dense), **I**. Mature autosporangium. Labels: (cw) cell wall, (n) nucleus, (pg) plastoglobuli, (s) starch granules, (th) thylakoids. Scale bars = 500 nm.

TEM observations revealed that the cell wall was smooth, formed by three distinct layers (Fig 3A) as observed in a *Coccomyxa* strain symbiont of the lichen *Solorina crocea* [52] and in some acidophilic strains of *Coccomyxa simplex*[53]. The parietal chloroplast was often cup-shaped, covering most of the inner cell wall surface (Fig 3B–3F). The chloroplast partially surrounded the nucleus (Fig 3D–3H) and filled approximately 2/3 of the total cell volume. It was devoid of pyrenoids and many starch granules were deposited in its interthylacoidal spaces (Fig 3B–3H). The nucleus was placed in the median part of the cell, usually slightly closer to a side (Fig 3G and 3H). TEM micrographs showed that in the cytoplasm the microalga contained several plastoglobuli filled with electron-dense material (Fig 3E–3H), which probably corresponded to lipid droplets observed in light microscopy (Fig 2G). In a young stage of autosporogenesis, daughter cells were already surrounded by relatively thick cell walls (Fig 3I).

### Molecular phylogeny

**Fig 4:**
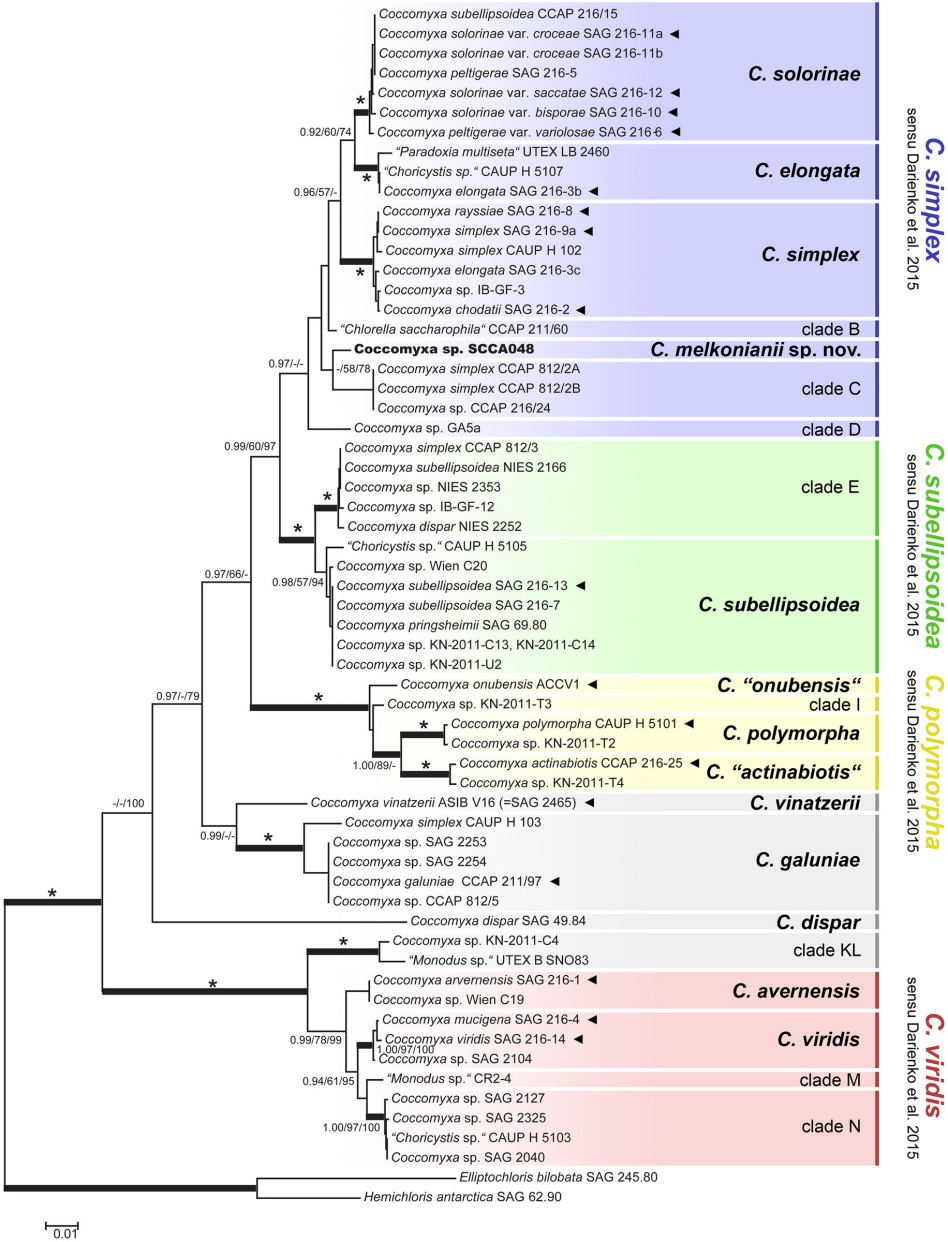
Phylogeny of the genus *Coccomyxa* obtained by Bayesian inference of the concatenated SSU and ITS rDNA dataset. Values at the nodes indicate statistical support estimated by three methods—MrBayes posterior-node probability (left), maximum-likelihood bootstrap (middle), and weighted maximum parsimony bootstrap (right). Full statistical support (1.00/100/100) is marked with an asterisk. Thick branches represent nodes receiving the highest PP support (1.00). The newly sequenced strain of *Coccomyxa* SCCA 048 is marked in bold. Sequences obtained from authentic strains are marked by arrowheads. Clades belonging to four species circumscribed by Darienko et al.[4], i.e., *C*. *simplex*, *C*. *subellipsoidea*, *C*. *polymorpha* and *C*. *viridis*, are marked in blue, green, yellow, and red, respectively. The clades recognized are either assigned to previously described *Coccomyxa* species or labelled alphabetically based on GMYC analyses (see Fig 5). Scale bar represents the expected number of substitutions per site.

The concatenated phylogenetic analysis of all available SSU-ITS rDNA sequences revealed the existence of a great number of well-supported lineages within the genus *Coccomyxa* (Fig 4). The tree topology corresponded well with the phylogram inferred by Darienko et al.[4], although our broader sampling resulted in the recognition of many novel lineages. Most of the analyzed sequences belonged to four species circumscribed by Darienko et al.[4], i.e., *C*. *simplex*, *C*. *subellipsoidea*, *C*. *polymorpha* and *C*. *viridis*. The newly isolated *Coccomyxa* sp. SCCA048 formed a distinct lineage within *C*. *simplex* s. l., along with the six additional lineages referred here to as *C*. *solorinae*, *C*. *elongata*, *C*. *simplex* s.str., clade B, clade C, and clade D. However, the monophyly of *C*. *simplex* s.l. obtained no statistical support in our analyses, and so the specific phylogenetic position of *Coccomyxa* sp. SCCA048 remained unresolved. *C*. *subellipsoidea* s.l. and *C*. *viridis* s.l. comprised two and four distinct lineages, respectively, as already recognized by Darienko et al.[4]. In addition, a sister lineage to *C*. *viridis* s.l. was recognized (clade KL), comprising *Coccomyxa* sp. KN-2011-C4 and “*Monodus* sp.” UTEX B SNO83. Three distinct lineages were additionally inferred within *C*. *polymorpha* s.l., including strains described as *C*. *actinabiotis* and *C*. *onubensis*, respectively. However, these species were not validly described so far due to the absence of a type designated.

### DNA-based species delimitation

We performed three DNA species delimitation analyses (GMYC, bPTP, STACEY) to estimate putative species boundaries in our *Coccomyxa* dataset. All analyses were run on four different datasets: i) a concatenated SSU + ITS rDNA alignment including all ITS nucleotide positions (62 taxa, 2689 bp), ii) a concatenated SSU + ITS rDNA alignment reduced to include only well-aligned ITS positions (62 taxa, 2311 bp), iii) an ITS rDNA alignment including all nucleotide positions (98 taxa, 906 bp), and iv) an ITS rDNA alignment reduced to include only well-aligned positions (98 taxa, 528 bp).

**Fig 5:**
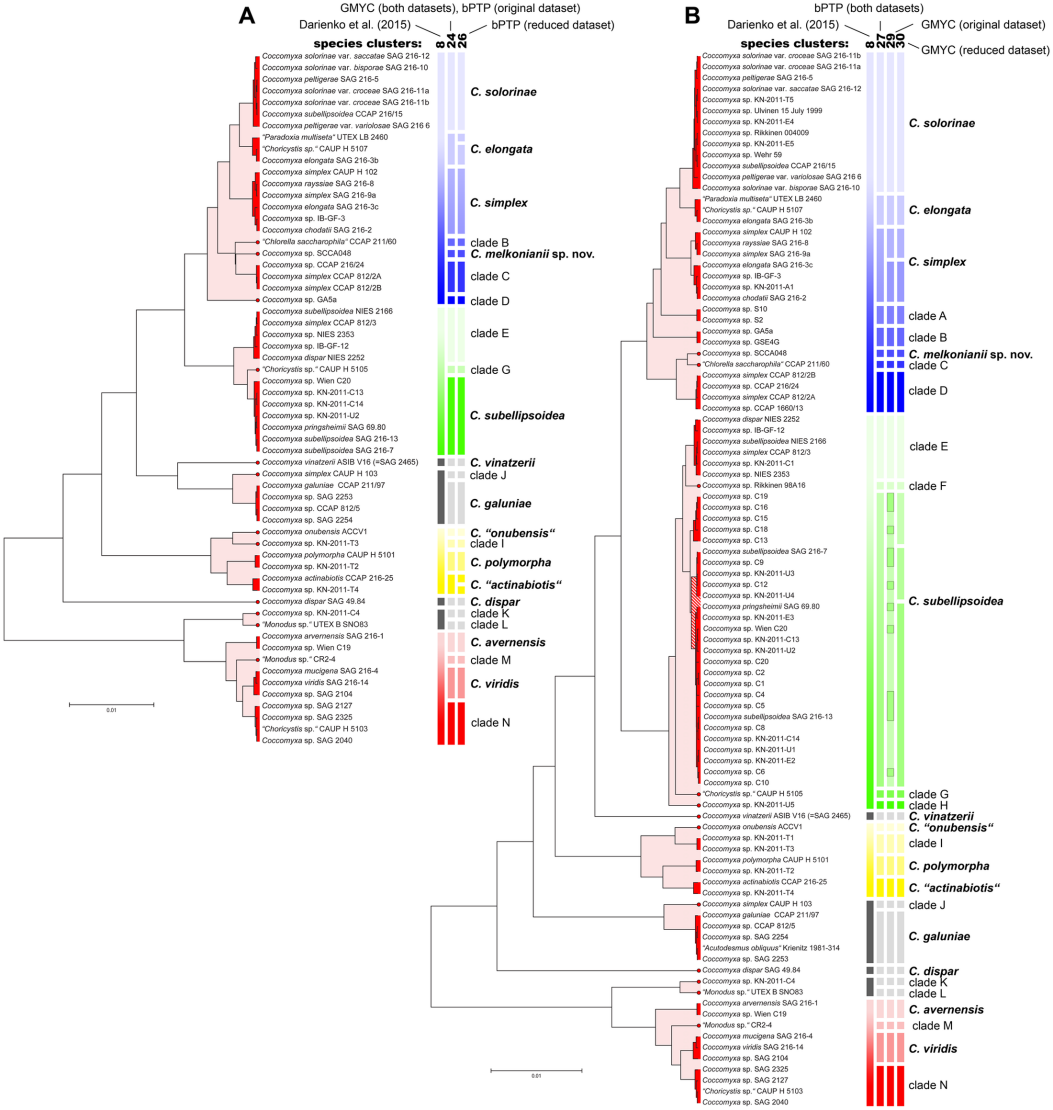
Comparison of different species scenarios resulting from GMYC and bPTP analyses, along with the species clusters proposed by Darienko et al.[4]. The topologies shown were obtained by BEAST analyses based on concatenated SSU + ITS rDNA (**A**) and ITS rDNA (**B**) datasets, using alignments reduced to include only well-aligned ITS positions. The red coloration delimits species clusters recognized by GMYC analyses; a hatched (red) pattern indicates the GMYC clusters containing genetically identical sequences. The pink coloration delimits the eight species clusters proposed by Darienko et al.[4]. Different species scenarios proposed by GMYC and bPTP analyses are indicated by vertical bars next to the phylogenetic trees. For each topology, two different datasets were analysed (including original and reduced ITS rDNA alignment to include only well-aligned ITS positions). The species clusters are either assigned to previously described *Coccomyxa* species or labelled alphabetically. Clade colouring follows the Fig 4. Scale bars represent the expected number of substitutions per site.

First, branch lengths in the ultrametric gene trees were analysed by single threshold GMYC analyses. The likelihoods of all four GMYC analyses were significantly higher than those of the null models of uniform coalescent branching rates (P values 0.00004, 0.00003, 0.00013, <0.00001, respectively). Both GMYC analyses based on the concatenated SSU + ITS rDNA datasets identically indicated the presence of 24 species clusters in *Coccomyxa*, with a narrow confidence interval obtained for the dataset with reduced ITS positions (23–28 *vs*. 17–31 species clusters). Accordingly, *C*. *simplex* s.l., *C*. *subellipsoidea* s.l., *C*. *polymorpha* s.l., and *C*. *viridis* s.l. were split into seven, three, four, and four distinct species clusters, respectively (Fig 5A). Twelve of 24 recognized species clusters can be assigned to previously described *Coccomyxa* species, based on the availability of authentic strains or morphologically well-studied specimens (Fig 4). The GMYC analyses based on the ITS rDNA datasets indicated the presence of 29 and 30 species clusters, respectively. The analysis based on the reduced ITS rDNA alignment recognized 30 species clusters (Fig 5B), with much narrow confidence intervals as compared to the unreduced dataset indicating 29 species (27–38 *vs*. 15–36 species clusters). The analyses differed in the clustering of species belonging to *C*. *subellipsoidea* s.str. Whereas the analysis based on the reduced ITS rDNA alignment split this species into the three clusters, the latter analysis recognized two species clusters (Fig 5B). However, both analyses erroneously indicated splitting of identical *C*. *subellipsoidea* sequences (*Coccomyxa* sp. C6, C8, C9, C10, C12) into distinct species clusters.

The two bPTP analyses on the concatenated SSU + ITS rDNA datasets recovered different species circumscriptions. The analysis based on the concatenated dataset with the original ITS rDNA alignment corroborated the species clustering proposed by the GMYC analyses, recognizing 24 clusters (Fig 5A): 20 of 24 species clusters were statistically highly supported, obtaining a posterior probability higher than 0.95. *C*. *“actinabiotis”*, *C*. *elongata* and *C*. *polymorpha* s.str. were among the most weakly supported scenarios (PP 0.78, 0.83 and 0.83, respectively). The analysis based on the concatenated dataset with the reduced ITS rDNA alignment recognized 26 species clusters, splitting two of these weakly supported species units (*C*. *elongata* and *“C*. *actinabiotis”*) into two clusters (Fig 5A). However, these clusters were also poorly supported (PP of both *C*. *elongata* and *C*. *“actinabiotis”* clusters was 0.86 and 0.53, respectively). The two bPTP analyses performed on the ITS rDNA datasets identically recovered the presence of 27 species clusters (Fig 5B). The composition of species clusters was identical to the 24-species scenario resolved by GMYC and bPTP analyses on the concatenated SSU + ITS rDNA dataset. The analysis based on the reduced ITS rDNA dataset performed well, with 25 of 27 species clusters being statistically highly supported (PP > 0.95). Among the most weakly supported species scenarios were *C*. *“actinabiotis”* and clade E (PP 0.74 and 0.88, respectively). However, the analysis based on the original ITS rDNA dataset performed much worse, with only 21 of 27 species clusters being statistically highly supported (PP > 0.95).

All the above-mentioned species scenarios were tested by the STACEY analyses to compare the statistical significance of species boundaries estimated by our GMYC and bPTP analyses with those proposed by Darienko et al.[4]. In general, most of the species scenarios were highly supported. The analysis on the concatenated SSU + ITS rDNA dataset preferred the 24-species scenario proposed by the majority of species delimitation analyses (PP 0.996) over the 8-species and 26-species scenarios (PP 0.991 and 0.948, respectively). The analysis on the ITS rDNA dataset revealed the 27-species scenario as being the most supported (PP 0.995), as compared to the 8-, 29-, and 30-species scenarios (PP 0.992, 0.950 and 0.779, respectively). Accordingly, the 24- and 27-species scenarios, proposing in fact the identical composition of species clusters, were selected as the best fitting models.

### Analyses of ecological data

To evaluate putative ecological differentiation of *Coccomyxa* lineages, we collected ecological data for the strains with sequences included in our ITS rDNA dataset. Then, we tested the strength of ecological differentiation of particular species, under five different species scenarios recognized by GMYC and bPTP analyses, as well as the 8-species scenario based on the conclusions of Darienko et al.[4]. The statistical significance of ecological differences among the putative species clusters was tested by chi-square and Mantel tests. The chi-square tests based on the 62-taxa dataset (testing the species scenarios based on the SSU + ITS rDNA data) indicated the significant differentiation of species under the 24- and 26-species scenarios, selecting the resilience and habitat type as the most important ecological traits (Table 2). However, Mantel tests did not identify any species clustering to be significantly correlated with ecological differentiation of investigated strains.

**Table 2:** Ecological differentiation of putative species clusters, based on the 62-taxa dataset.

Statistical test	Species scenario	Ecological traits		
		Living stage	Resilience	Habitat type
**χ**^**2**^	8 species	0.428	0.087	0.075
	24 species	0.149	**0.003**	**0.013**
	26 species	0.267	**<0.001**	**0.010**
**Mantel test**	8 species	0.111	0.075	0.063
	24 species	0.117	0.853	0.353
	26 species	0.207	0.938	0.462

Three different species scenarios were evaluated by chi-square and Mantel tests, evaluating the differences in living stage (free living, lichen photobionts, ciliate symbionts), resilience (mesophilic, extremophilic), and habitat (water, soil, rock, bark). Significant P values are given in bold.

The chi-square tests based on the 98-taxa dataset (testing the species scenarios based on the ITS rDNA data) indicated the significant ecological differentiation of species under the majority of species scenarios, over all tested ecological traits, with the exception of the 8-species scenario where only habitat type significantly differentiated the species clusters (Table 3). On the other hand, Mantel tests clearly selected the 27-species scenario as the only one showing significant ecological differentiation of species clusters. The species were primarily differentiated by their living stage, so the free living algae, lichen photobionts, and ciliate symbionts significantly clustered into the 27 species.

**Table 3:** Ecological differentiation of putative species clusters, based on the 98-taxa dataset.

Statistical test	Species scenario	Ecological traits		
		Living stage	Resilience	Habitat type
**χ**^**2**^	8 species	0.034	0.011	**<0.001**
	27 species	**<0.001**	**<0.001**	**<0.001**
	29 species	**<0.001**	**<0.001**	**<0.001**
	30 species	**<0.001**	**<0.001**	**<0.001**
**Mantel test**	8 species	0.695	0.863	0.147
	27 species	**<0.001**	0.815	0.059
	29 species	0.054	0.807	0.652
	30 species	0.157	0.779	0.737

Four different species scenarios were evaluated by chi-square and Mantel tests, evaluating the differences in living stage (free living, lichen photobionts, ciliate symbionts), resilience (mesophilic, extremophilic), and habitat (water, soil, rock, bark). Significant P values are given in bold.

**Fig 6:**
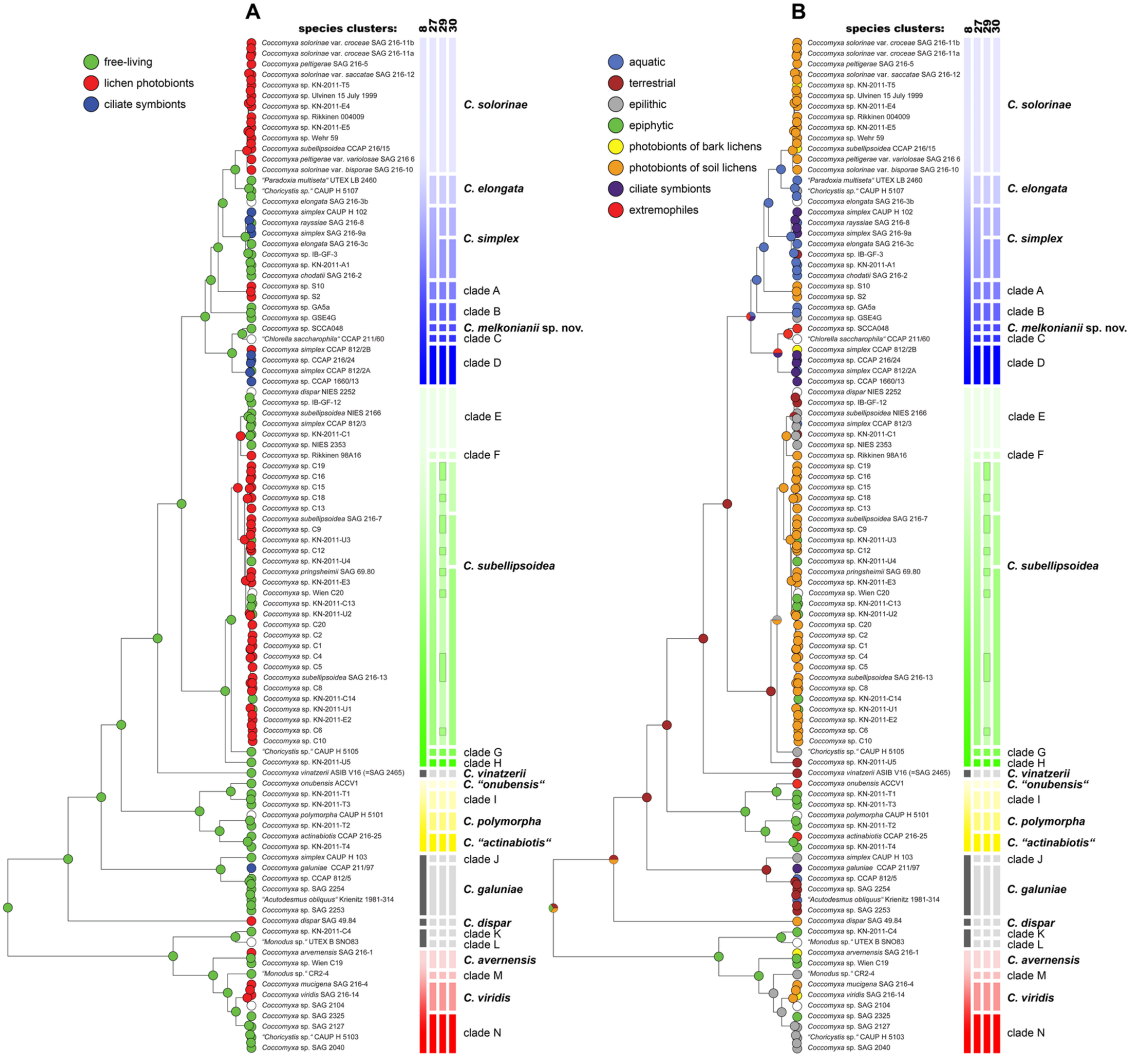
Estimated evolution of ecological traits within the genus *Coccomyxa*. Ancestral reconstructions of the living stage (**A**) and habitat ecological characteristics (**B**) were inferred using the ACCTRAN parsimony method. The topologies shown were obtained by BEAST analyses based on a concatenated SSU + ITS rDNA dataset, using alignments reduced to include only well-aligned ITS positions. Circles at terminal nodes represent the observed trait state for extant species. Pie charts for ancestral nodes show estimated proportions for reconstructed trait states at that internal node. Different species scenarios proposed by GMYC and bPTP analyses are indicated by vertical bars next to the phylogenetic trees. Clade colouring and labelling follows the Figs 4 and 5.

Summarizing our results, statistical tests showed narrowly defined species to be significantly better delimited by their ecology than the broadly delimited species, indicating the 27-species scenario to be the most consistent with ecological data. Indeed, a number of narrowly defined species can be well characterized by various ecological traits (Fig 6). Differences in living stage can be well applied to delimit species within the *C*. *simplex* s.l. (Fig 6A): *C*. *solorinae* and clade A represent lichen photobionts, whereas *C*. *elongata* and clade B include free living strains only. In addition, *C*. *simplex* s.str. and clade D comprise free-living species often entering into symbiotic association with the heterotrophic ciliates. Further ecological differentiation of narrowly defined species can be revealed when considering habitat-related traits (Fig 6B). For example, the terrestrial strains generally clustered in *C*. *galuniae* and the clade E. However, whereas the former species can be also found in aquatic environment, the latter one is strictly aerophytic. Within *C*. *simplex* s.l., the association with an extreme environment (a river with very high contents of heavy metals) was exclusive of *Coccomyxa* SCCA048, hereafter described as *Coccomyxa melkonianii* sp. nov.

## Discussion

### Re-evaluation of the species concept in *Coccomyxa*

Traditionally, species of the genus *Coccomyxa* have been differentiated by morphological features, such as cell shape and dimensions, structure and distribution of mucilaginous sheaths, and number of autospores [26–28]. In the first comprehensive monograph of the genus, Jaag [26] summarized the information available at the time on the diversity of *Coccomyxa*, and delimited a total of 14 free-living species, 6 taxa of lichen epiphytes, and 13 lichenized taxa. The species belonging to the genus, including those newly described in this monograph, were delimited based primarily on the structure of mucilaginous colonies and detailed investigations of cell length and width variability. In a more recent morphological assessment of *Coccomyxa*, Ettl & Gärtner [28] distinguished only seven well-recognized species, based on differences in cell shape and size, number of chloroplasts and mucilage properties. These authors considered inadequately delimited most of the species known at the time, which were defined using only morphological features.

Since the description of the genus, the relevance of particular morphological traits to delimit *Coccomyxa* species was often discussed and re-evaluated [28]. For example, cell shape and dimensions were proven to be significantly dependent on culture conditions [26]. Darienko et al. [4]demonstrated significant phenotypic plasticity in several strains grown at different salinities. The presence of mucilaginous sheaths, traditionally used to separate the genera *Coccomyxa* and *Pseudococcomyxa*, has been shown to be highly dependent on nutrient availability. The problematic morphological delineation of *Coccomyxa* species is well demonstrated by the fact that some strains of this genus deposited in culture collections were erroneously determined as members of the morphologically similar genera *Chloroidium* (= *Chlorella saccharophila*), *Choricystis* and *Monodus* (Fig 4).

Given the limited value of morphological data for species circumscription in *Coccomyxa*, Darienko et al. [4]proposed to separate taxa using several DNA-based delimitation approaches. Accordingly, these authors subdivided the genus in 7 distinct species, including the broadly defined C. *simplex* s.l., *C*. *subellipsoidea* s.l. and *C*. *viridis* s.l. In this study we have followed a similar approach, but for the phylogenetic analyses we have analyzed an expanded dataset that includes 18 additional SSU + ITS rDNA sequences, publicly available but not used by Darienko et al.[4] for their analyses. Our DNA-based and ecological analyses draw a rather different scenario, favouring a split into 27 species much more narrowly defined. Based on these results, we propose an alternative species circumscription for *Coccomyxa*, in which ecological characters play a major role for species delimitation. With the support of our analyses of ecological data, we argue that any well-resolved phylogenetic lineage which is ecologically distinct from its relatives should be regarded as a separate species.

Therefore, we propose to consider every well-resolved, ecologically distinct lineage within *C*. *simplex* s.l. as separate species as follows: *C*. *solorinae* as a lineage of lichen-forming and free-living algae never growing on rocky surfaces; *C*. *elongata* as a species living in freshwater habitats and on wet sandstone rocks; *C*. *simplex s*.*str*. as a freshwater species capable to establish symbiotic interaction with ciliates; and a lineage formed by our isolate SCCA048 as an extremophile species capable to grow in high concentrations of heavy metals [54]. Accordingly, we describe here this lineage as a new species, *C*. *melkonianii*.

#### *Coccomyxa melkonianii* Malavasi et Škaloud sp. nov. (Figs 2 and 3)

**Diagnosis**: Vegetative cells narrowly ellipsoidal and slightly asymmetrical, 3–5.4 wide and 6–8.5 μm long, regularly curved, with rounded apices and without mucilaginous sheath. Cell wall smooth, with a median depression, trilaminar in ultrastructure. Chloroplast parietal, cup- shaped, covering much of the inner cell wall surface, with starch granules within the interthylacoidal spaces and without pyrenoid. One nucleus positioned in the central part of the cell. Protoplast filled with lipid droplets. Asexual reproduction by 2–4 autospores released by apical splitting of the mother cell wall. Extremophilic alga, living in fresh waters contaminated by high amounts of heavy metals. It differs from other species of *Coccomyxa* in SSU rDNA and ITS rDNA sequences.

**Holotype**: The strain CAUP H 104 permanently cryopreserved in a metabolically inactive state (cryopreservation in liquid nitrogen) in CAUP, Benátská 2, Praha 2, Czech Republic. The living culture (ex-holotype) has been deposited as SCCA048 at the University of Cagliari, Interdepartmental Center of Environmental Science and Engineering (CINSA)[55].

**Type locality**: Rio Irvi river, SW Sardinia, Italy (N 39° 32' 48.82'', E 8° 28' 49.08'').

**Etymology**: The species is named in honour of Prof. Michael Melkonian, an authority in phycology. He inspired the first author to study microalgae and to create the Culture Collection for the Sardinia region (Sardinian Culture Collection of Algae; SCCA).

**Authentic strain**: SCCA048

In addition to *C*. *melkonianii*, our 27-species scenario supports the recognition of several lineages that at the moment do not have a specific attribution. Among them, two lineages of extremophilic strains belonging to *C*. *polymorpha* s.l. have been proposed to represent separate species, namely *C*. *actinabiotis* and *C*. *onubensis*[56,57]. However, both species have been invalidly published as neither formal diagnosis was provided nor the holotypes were indicated at the time of publication. Since the descriptions are basically inexistent, it will be necessary to re-describe them with different names providing diagnoses and typification. Although a full taxonomic reassessment of *Coccomyxa* is necessary, we believe that description of any of these newly recognized lineages would be premature. *Coccomyxa melkonianii* is the only strain that we collected and for which we could directly do an accurate morphological and molecular study. The description of new species for the lineages that at the moment do not have a specific attribution in our opinion should be based on similar observations.

### What is the best way to delimit species of microalgae?

The results of this study have some important implications that extend well beyond the genus *Coccomyxa* and apply to many other taxa of microalgae. Systematics and species delimitation of microbial eukaryotes (protists) have been discussed in many recent publications and are still highly controversial matters (Ryšánek et al. [58]and references therein). Much of the controversy on these topics is related to the criteria used for the circumscription of protist species and the presumed patterns of speciation in these organisms.

The use of DNA sequence data for species identification and circumscription has been seen as a convenient solution to the problems affecting other types of data, and has now more than ten years of history [59]. During the last decade, there has been much use of DNA sequence variation data at or near species level to explore the relationships among closely related species, to date species ages, or to delimit species [60]. In recent years, the number of methods available for delimiting species based on sequence data has dramatically increased (see review in Carstens et al.[18]). Some methods, in particular those based on assessment of distances between sequences (*e*.*g*., ABGD, Puillandre et al.[17]) and on the coalescent theory (*e*.*g*., GMYC, Brownie, SpedeSTEM, BPP; [20,21,18,61]) have gained great popularity and have been applied to many taxonomic groups [62–64]. The goal of these methods is to provide a convenient and reliable tool for species delineation; they have been used in many studies as a base to justify taxonomic conclusions, particularly the split into numerous species of difficult taxa for which morphological discrimination is impossible (*e*.*g*., [65–68]). However, it is also acknowledged that in some cases these analyses have the potential to provide misleading results [60], which may complicate considerably the recognition of species boundaries and relationships. These methods have some assumptions that are not always met in the databases analyzed; it is well known that some of the most used, such as GMYC, are highly dependent on taxon sampling and may underperform with datasets containing a high number of rare species, that cannot be sampled densely [18,62,69]. Cases of erroneous delimitation of species boundaries have been reported for cryptomonads [62], gastropods [70], frogs [71], bats [72] and chimpanzees [73]; in particular, the recognition of multiple species within groups formed by identical sequences is a problem that has been documented in some studies [62] and that we encountered in some of our analyses (*e*.*g*., identical sequences of *Coccomyxa subellipsoidea* split into several species in the GMYC analysis of the original ITS dataset).

Carstens et al. [18]remark that inferences regarding species boundaries based on genetic data alone are likely inadequate and species delimitation should be conducted with consideration of life history, geographical distribution, morphology, and behaviour; these authors also recommend that DNA-based species circumscriptions should use several different methods and be based on multi-locus taxon sampling of numerous samples for each putative lineage. We agree with these considerations and we note that in this regard special caution is required when dealing with eukaryotic microalgae. Eukaryotic microalgae are a highly heterogeneous assemblage formed by many groups of organisms with different origins and evolutionary histories. In some groups, particularly in green microalgae, some of the conditions required for a robust species delimitation are often difficult to satisfy. New species of microchlorophytes continue to be described at steady rate (*e*.*g*., [2,12,74–78]), sometimes even in groups in which the taxonomy is presumptively consolidated [79,80]. This suggests that the real species diversity for many groups of microchlorophytes is not yet adequately known, and therefore it is difficult to understand how reliable DNA delimitation analyses may be for these algae. Even if the overall diversity of a group is well assessed, achieving a good sampling of species is often difficult in protistan lineages [62], due to the small size, difficulty of collection in the field and, for some lineages, impossibility of maintenance in culture. The characteristics and the mode of evolution of the molecular marker used is another aspect that should be kept in consideration especially in coalescence-based analyses, as the use of markers with different coalescence rates (for example, nuclear markers versus organellar markers) may lead to different species circumscriptions. In consideration of these facts, it is not surprising that our analyses led to a substantially different scenario from the taxonomic arrangement of *Coccomyxa* proposed by Darienko et al.[4]; in all our analyses, a subdivision in a much higher number of species (24 or 27) was consistently better supported than the 8-species scenario suggested by Darienko et al.[4]. Our results should be seen as an advancement, rather than a rejection of the conclusions of Darienko et al.[4], and highlight the importance of an extensive taxon sampling; we find remarkable that such a different outcome was determined by the addition of a relatively modest number of new sequences (about one third more of the sequences used by Darienko et al.[4]). Since the genus *Coccomyxa* is cosmopolitan and inhabits an exceedingly wide range of habitats, we believe it to be unlikely that the data currently available represent a full coverage of the diversity of this genus. We expect that new lineages of *Coccomyxa* will be discovered in the near future and that future analyses for species delimitation will lead to different outcomes from the scenario drawn by our results, possibly with a much higher number of species. In addition, sequencing of additional molecular markers would certainly increase the quality of species delimitation analyses, allowing us to use the promising multi-locus approaches [16]. In any case, an integrative approach combining genetic data with a detailed knowledge of phenotypic traits remains in our opinion imperative. We suggest that, among phenotypic traits, ecological characters should be given special emphasis.

Ecological characters have been used for species delimitation both in animals (*e*.*g*., [81,82,83]) and, particularly, in plants [84–87], sometimes based on accurate ecological niche modelling analyses. In the case of microalgae, ecological features have generally not received similar consideration, mainly because of the difficulty of assessing the distribution of microscopic organisms in the field. However, recent studies combining morphological and ecological data support more and more the possibility that sympatric differentiation driven by different ecological preferences may lead to the origin of different lineages, and eventually different species. For example, different lineages of the cosmopolitan genus *Klebsormidium* differ in the type of substratum colonized [11,88], pH preferences [89–91] or distribution in climatic areas [92]. Different lineages of the lichen photobiont *Asterochloris* exhibit clear differences in terms of environmental preferences, being associated with lichens adapted to different rainfall and sunlight exposure [93]. Some species of *Trebouxia*, a genus closely related to *Asterochloris*, are strictly associated with localities with specific microclimates [13]. Closely related species of the genus *Prasiola* differ in terms of aquatic/terrestrial habitat [10,94]. In the subaerial genus *Trentepohlia*, closely related species differ in terms of substratum occupied, with some distributed on tree bark and others on limestone or artificial substrata [95]. Unfortunately, the details of speciation processes in these organisms are still poorly understood and it is difficult to make generalizations about the exact mechanisms and rate of origin of new lineages; in the case of *Klebsormidium*, Škaloud and Rindi [11] suggested that selective sweep combined with the selection of new mutants differing in ecological niche may have played a major role in the diversification of the genus. Green microalgae are currently the target of intensive genomic work and there is no doubt that genomic data will shed light into the mechanisms that drive ecological differentiation and origin of new species. For the moment, we suggest that, when closely related lineages distinguishable by ecological preferences are well supported in molecular phylogenetic analyses, they should be recognized as different species regardless of ambiguities in other types of data. This is a situation that fits well with the 27-species scenario, that was the best supported by our analyses for species delimitation in *Coccomyxa*. Our tests indicated significant ecological differentiation of species under the majority of species scenarios, with a particularly clear effect of living stage in the 98-taxa dataset (Table 3). The narrowly defined circumscription allows an ecology-based definition of species in the clade of *C*. *simplex* s.l., where *C*. *melkonianii* represents the only lineage associated to a habitat with extreme features (very high concentrations of heavy metals; Fig 6B). This species represents yet another example in support of the idea that extreme environments promote evolution and speciation in green microalgae, a hypothesis that has already received support by other studies focusing on different environments [12,88,96,97].

## Conclusions

A great number of species concepts have been proposed so far, ranging from the traditional morphological concept to various modern DNA-based delimitation concepts. It is generally accepted that in the case of green microalgae there is not a single species concept that is universally applicable, and the results of this study are in agreement with this conclusion. The modern methods for DNA-based species delimitation represent a powerful tool that has proven useful in many studies focusing on a broad range of organisms. They are, however, very sensitive to data sampling, and must be applied with great caution in studies on microbial eukaryotes, particularly in groups where the genetic diversity is incompletely sampled. Only a precise taxonomic work combining various methodological approaches can determine the species boundaries correctly, and can help us to organize the natural diversity in a meaningful fashion. Ecological data can represent a very useful tool to delimit morphologically often undistinguishable species, and we strongly recommend that ecological characterization of microalgae should receive much more attention than at present.

## Supporting Information

S1 Dataset Multiple alignment of 98 concatenated SSU rDNA and ITS rDNA sequences, aligned using the MAFFT v. 6 software and ITS secondary structures. (FAS)

S2 Dataset Multiple alignment of 62 concatenated SSU rDNA and ITS rDNA sequences used to infer the *Coccomyxa* phylogeny. Poorly aligned positions were eliminated using the program Gblocks v. 0.91b.
(FAS)

S3 Dataset Multiple alignment of 62 concatenated SSU rDNA and ITS rDNA sequences used in DNA-based species delimitation analyses. The original, not-trimmed ITS rDNA alignment of 917 nucleotide positions is used.
(FAS)

S4 Dataset Multiple alignment of 62 concatenated SSU rDNA and ITS rDNA sequences used in DNA-based species delimitation analyses. The reduced ITS rDNA alignment of 528 nucleotide positions is used. Ambiguously aligned positions were eliminated by the program Gblocks.
(FAS)

S5 Dataset The original, not-trimmed ITS rDNA alignment used in DNA-based species delimitation analyses. (FAS)

S6 Dataset The reduced ITS rDNA alignment used in DNA-based species delimitation analyses. Ambiguously aligned positions were eliminated by the program Gblocks.
(FAS)

S1 Table List of sequences analyzed in this study. Classification, accession numbers, affiliation to different alignment datasets, and ecological data are provided.
(XLSX)
